# LGI1-antibody associated epilepsy successfully treated in the outpatient setting

**DOI:** 10.1016/j.jneuroim.2020.577268

**Published:** 2020-08-15

**Authors:** R. Uribe-San-Martín, E. Ciampi, R. Santibañez, S.R. Irani, A. Márquez, J.P. Cruz, B. Soler, M.C. Miranda, M. Henríquez, C. Cárcamo

**Affiliations:** a Neurology Service, Hospital Dr. Sótero del Río, Santiago, Chile; b Neurology Department, Pontificia Universidad Católica de Chile, Santiago, Chile; c Oxford Autoimmune Neurology Group, Nuffield Department of Clinical. Neuroscience, John Radcliffe Hospital, University of Oxford, Oxford, UK; d Department of Neurology, Oxford University Hospitals NHS Foundation Trust, Oxford, UK; e Neuroradiology Department, Pontificia Universidad Católica de Chile, Santiago, Chile.; f Clinical Laboratories Department, Pontificia Universidad Católica de Chile, Santiago, Chile.

**Keywords:** Leucine-rich glioma-inactivated 1 (LGI1) autoantibodies, Antibody mediated seizures, Autoimmune epilepsy, Carbamazepine, Neuroimmunology

## Abstract

We report six patients with anti-LGI1 associated epilepsy. Two patients presented with new-onset generalized tonic-clonic seizures, four developed faciobrachial dystonic seizures and two piloerection. All patients had significant cognitive complaints at the time of diagnosis. All patients described seizure reduction during the first week of carbamazepine, and seizure freedom was obtained at a median of 13 days (range 7–22), sustained after the initiation of immunosuppression. Median time from symptom onset to carbamazepine initiation was 164 days (range 38–206 days). We discuss the particular seizure response to sodium channel blocking antiepileptic drugs, alone or associated with immunosuppression in this antibody mediated seizures.

## Highlights

• Anti-LGI1 associated epilepsy may particularly respond to sodium channel blocking AED.
• Immunosuppression is usually needed to achieve sustained seizure freedom (SzF).
• Carbamazepine and prednisone are affordable and available in developing countries.
• We report 6 patients with SzF within two weeks of carbamazepine +/− immunotherapy.
• Treatment was well tolerated and seems cost-effective.

## Graphical abstract

**Figure:**
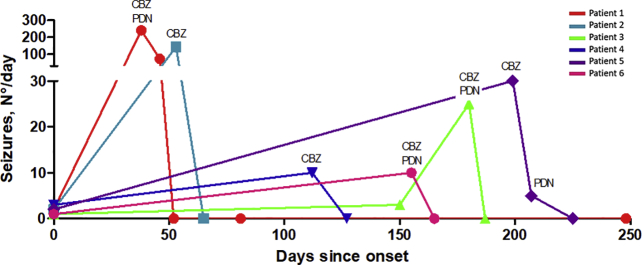

## 1 Introduction

Leucine-rich glioma-inactivated 1 (LGI1) protein is expressed predominantly within the hippocampus and involved in the function of the presynaptic voltage-gated potassium channels and postsynaptic α-amino-3-hydroxy-5-methyl-4-isoxazolepropionic acid (AMPA) receptors. Its mutation is a known cause of autosomal dominant lateral temporal lobe epilepsy (Varley et al., 2018).

In 2010, Irani et al. (Irani et al., 2010) described for the first time the antibodies directed against LGI1. This finding characterized a clinical spectrum of limbic encephalitis, with generalized seizures and cognitive impairment. In some patients, a particular type of seizure, faciobrachial dystonic seizures (FBDS) is seen. FBDS present with frequent episodes during the day, with only a few showing electroencephalographic correlates. Several other seizure semiologies have been described which are strongly associated with LGI1 antibodies. These include ictal bradycardia (Naasan et al., 2014) and piloerection (Rocamora et al., 2014). Nowadays, some these seizures are considered essentially pathognomonic of LGI1-antibody encephalitis, with a consistent response to immunosuppressive treatment (Irani et al., 2008, Irani et al., 2011).

Recent reports in patients with LGI1-antibodies describe a preferential clinical response to sodium channel blocking antiepileptic drugs, in particular to carbamazepine and lacosamide, used alone or alongside immunosuppression (Feyissa et al., 2017; de Bruijn et al., 2019). Although rituximab has been described as an effective therapy (Irani et al., 2014), in developing countries there is less access to inpatient infusion services and, hence, monoclonal antibodies. Therefore, effective low-cost outpatient treatment protocols using carbamazepine complemented with oral immunosuppression, are potentially very appealing for combining pragmatism with seizure control.

We present a case-series highlighting the clinical presentation and outcomes of six patients with LGI1-antibody mediated seizures, diagnosed and treated mainly in outpatient setting with carbamazepine and prednisone. We also describe their paraclinical findings and neuropsychological profiles.

## 2 Material and methods

### 2.1 Included patients

A prospective case-series was reviewed of consecutive patients admitted in the Neurology Services in the Pontificia Universidad Católica and Hospital Dr. Sótero del Río from January 2018 to April 2019. We collected clinical findings, paraclinical and immunology laboratory tests, electroencephalogram (EEG) and Magnetic Resonance Imaging (MRI) performed during the diagnostic process. Patients with missing data (e.g. family history or precipitants) were telephoned for data accrual. Treatment protocols, response to treatment and adverse events were also collected during the following visits.

### 2.2 Autoimmune encephalitis panel

Antibody detection was performed in patient's sera and cerebrospinal fluid (CSF) at the dependencies of the Service of Clinical Laboratories UC-CHRISTUS, accredited by ISO 15189. Briefly, the Autoimmune Encephalitis Panel in this laboratory consists in the detection of LGI1, CASPR2 (contactin-associated protein 2), *N*-methyl-D -aspartate receptor (NMDAR), AMPA receptor 1 and 2, and γ -amino-butyric acid receptor (GABA B R) by indirect immunofluorescence, using commercial available biochip mosaics containing as antigenic substrates formalin-fixed HEK293 cells transfected with the aforementioned neuronal surface protein antigens. An additional evaluation of frozen sections of rat hippocampus and rat cerebellum is also included in order to detect autoantibodies directed towards targets not specifically expressed on the cell-based assays. Each immunofluorescence pattern is evaluated independently by a trained technologist and a board-certified pathologist.

## 3 Results

### 3.1 Demographics

From January 2018 to April 2019, 6 patients with LGI1-antibody mediated seizures were diagnosed, 3 women, mean age 55.8 years (median 53.0; range 44–75). The median time of diagnostic delay was 164 days (range 38–206). All patients had LGI1 antibodies in serum; CSF was studied in 3 patients with 1 being positive for LGI1. No other antibodies were found in the autoimmune encephalitis panel, serum or CSF. After a median follow-up of 15 months (range 10–22), no tumour or other systemic autoimmune disorders were diagnosed in these patients.

### 3.2 Features at onset

**Fig. 1:**
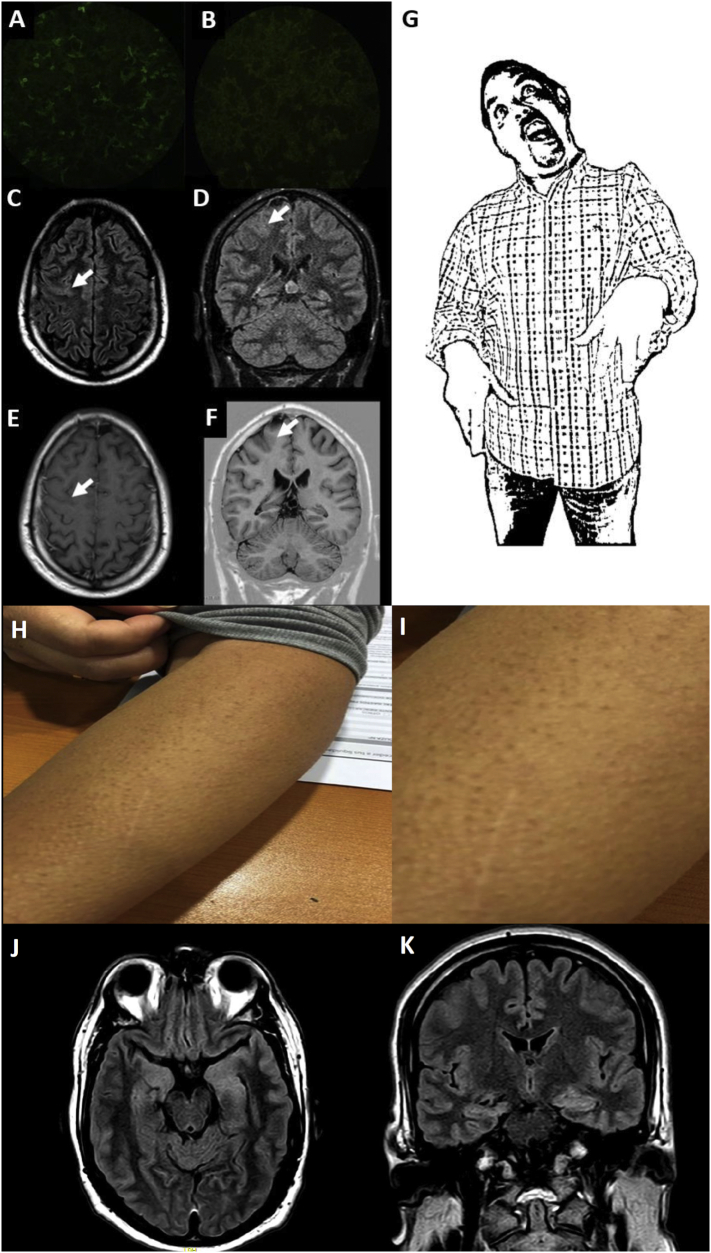
Clinical and paraclinical findings. Patient 1. LGI1 immunofluorescence positive in serum (A) and negative in cerebrospinal fluid (B). Magnetic Resonance Imaging (C) Axial and (D) Coronal FLAIR showing mild cortical thickening and increased T2 signal of the posterior aspect of the middle frontal gyrus (E) CE-Axial T1 shows no parenchymal or leptomeningeal enhancement. (F) Coronal T1W Inversion Recovery (arrows) shows no blurring of the grey/white matter junction, so a cortical dysplasia was considered unlikely. No hippocampal hyperintensity or atrophy was seen (not shown). Drawing of Faciobrachial dystonic seizures (G). Patient 5 selected pictures of piloerection seizures (H), zoom (I). Patient 6. Axial (J) and coronal (K) FLAIR MR Images show mild asymmetric enlargement of the both temporal unci and hippocampi head (mesial temporal region).

Two patients presented with new onset generalized tonic-clonic seizure (GTCS). Four patients presented with typical bilateral FBDS, with a predominance of one side. Median seizure frequency was 27.5 per day (range 10–240). One patient developed bilateral tonic-clonic seizures during follow-up. Patient 1 and 2, had some FBDS triggered by postural changes, such as rising from a chair and walking. Patient 3 presented her first GTCS two months after she received the yellow fever vaccine. Patient 4 started with sensitive focal seizures on his left side, some of them associated with piloerection, that were misinterpreted as meralgia paresthetica until he presented a focal impaired awareness. Patient 5 presented with bilateral predominantly left sided piloerection, approximately 30 times per day. Patient 6, began with lower extremities myalgias and memory complaints, then focal impaired awareness, then bilateral tonic-clonic seizures followed by FBDS, and finally, he presented with lower extremities fasciculations with electromyographic correlate, in the context of negative CASPR2 antibodies. Although all the patients developed seizures during follow-up, the initial complaint was confusion and dizziness in Patient 1, amnestic syndrome in Patient 4, and lower extremities myalgia in Patient 6. Representative seizures and paraclinical data are shown in Fig. 1, and a summary of patients' characteristics and time to treatment response is described in Table 1.

**Table 1:** Characteristics of patients with LGI1 autoimmune epilepsy.

Patient	1	2	3	4	5	6
Gender	M	F	F	M	F	M
Age at disease Onset	44	75	53	61	53	49
Presenting complaint	confusion	seizures	seizures,	amnesic syndrome	seizures	lower extremities myalgia and fasciculations
Seizure type at onset	2 GTCS (same day of presentation)	focal impaired awareness	GTCS	sensitive - piloerection	bilateral piloerection	focal impaired awareness
Seizure type during follow-up	FBDS	Temporal Lobe – FBDS	FBDS	Sensitive FS Piloerection	Piloerection	Temporal lobe BTCS FBDS
Max Seizures per day	240	144	25	10	30	10
First AED	same day LZP, PHT	LEV	LEV	PGB	–	LEV
Time to first AED (days)	0	30	30	10	–	132
Second AED	VPA, CLN	CBZ	CBZ	–	–	–
Time to second AED (days)	7	–	180	–	–	–
Third AED	LEV	–	CLB	–	–	–
Time to third AED (days)	19	–	201	–	–	–
Time to CBZ (days)	38	53	180	112	199	155
CBZ Doses (mg/d)	600	100	600	600	400	600
Time to Prednisone / immunoterapy (days)	38	–	180	188	207	155
Prednisone Doses (mg/d)	60	–	60	60	60	60
Time to IVPM (days)	–	58	30	–	–	–
Time to RTX (days)	–	61	–	–	–	–
Time to AZA (days)	–	–	194	–	–	–
Time to Seizure freedom (days)	52	65	187	127	225	165
EEG	Bilateral Frontal Slow Wave	Normal	Normal	Normal	Normal	Normal
MRI	Left pre- central sulcus	Normal	Normal	Right mesial temporal	Right mesial temporal	Normal
LGI1 Serum/CSF	+/−	+/UR	+/UR	+/−	+/UR	+/+
Allergy	–	–	Steven Johnson after 3 weeks of CBZ prescription and 3 iodinated contrast angiograms, change to CLB + LEV	–	–	rash 4 weeks after CBZ initiation, change to OXC + LCM

LGI1 Leucine-rich glioma-inactivated 1, AED antiepileptic drug, EEG electroencephalogram, MRI magnetic resonance imaging, CSF cerebrospinal fluid, CBZ carbamazepine, mg/d milligrams per day, M male, F Female, GTCS generalized tonic clonic seizures, BTCS bilateral tonic-clonic seizures, FBDS faciobrachial dystonic seizures, PHT phenytoin, VPA valproic acid, CLN clonazepam, LEV levetiracetam, CBZ clobazam, OXC oxcarbazepine, PGB pregabalin, LTG lamotrigine, LCM lacosamide, MTP methylprednisolone, RTX rituximab, PDN prednisone.

### 3.3 Cognitive performance

All patients developed cognitive complaints during the course of the disease. Four patients had a brief cognitive assessment, and two of those four had at least one follow up visit. All four patients reported short-term memory impairment early during the development of the disease. The severity of the memory impairment reported ranged from subjective complaints to impairment severe enough to affect instrumental activities of daily living. History from a family member was obtained in all four cases, and short-term memory problems were noted as the main abnormalities in all subjects. Other cognitive symptoms informed were spatial disorientation in one subject and inattention in another. Neuropsychiatric symptoms were also reported in three subjects, including irritability, disinhibition, and insomnia.

On cognitive testing using the Montreal Cognitive Assessment and the Frontal Assessment Battery, three patients exhibited delayed recall difficulties. The same three patients also showed a good response to cues. Another common abnormality noted on cognitive testing was mild executive dysfunction. Remarkably, all patients reported slowly progressive improvement in memory, neuropsychiatric symptoms, and overall functionality after starting therapy. Additionally, improvement in cognitive performance was observed in the two cases who had cognitive re-assessment on follow-up visits. Supplementary Table shows the cognitive performance at baseline and follow-up.

### 3.4 Treatment protocol

**Fig. 2:**
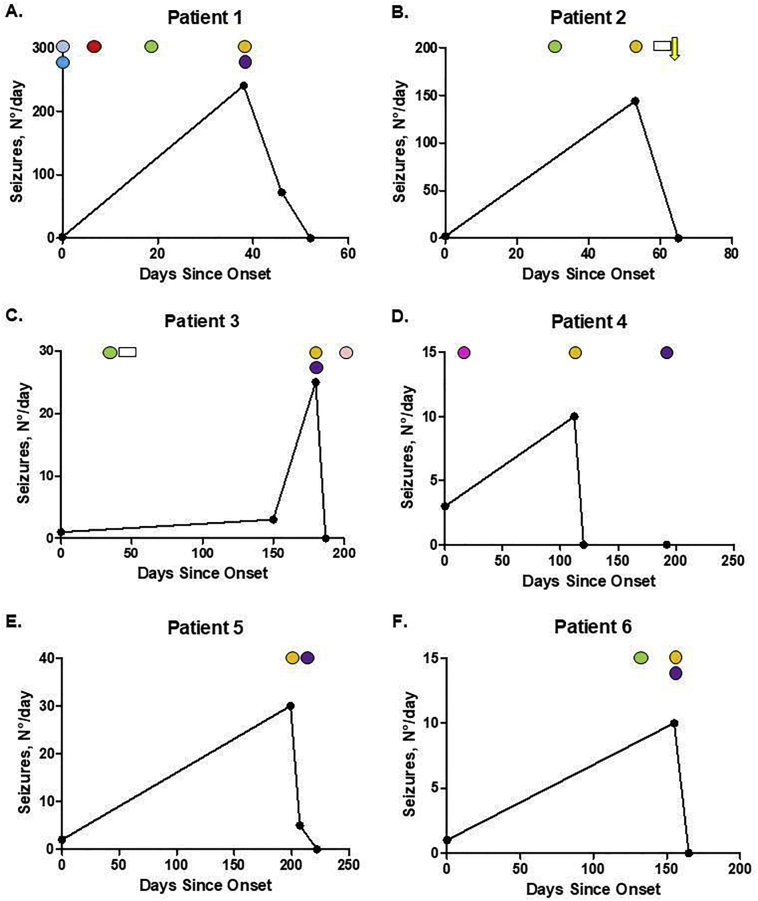
Treatment response. The graph represents seizure response after initiation of antiepileptic drugs and immunotherapy. N°/day number of seizures per day. Cyan circle: lorazepam, light blue circle: phenytoin, red circle: valproic acid, green circle: levetiracetam, orange circle: carbamazepine, purple circle: oral prednisone, white rectangle: intravenous methylprednisolone, yellow arrow: rituximab, pink circle: clobazam, magenta circle: pregabalin. (For interpretation of the references to colour in this figure legend, the reader is referred to the web version of this article.)

Seizure frequencies of the individual patients are summarised in Fig. 2. The median time from symptom onset to carbamazepine initiation was 164 days (range 38–206 days), and the median time from symptom onset to steroids (oral or intravenous) was 107 days (range 30–207). One patient received immunosuppression before carbamazepine without substantial seizure response (Patient 3), and one patient achieved seizure freedom after two weeks of carbamazepine without prednisone (Patient 4). All the other patients described seizure reduction during the first week of carbamazepine plus immunotherapy, and seizure freedom was obtained after a median of 13 days of starting carbamazepine (range 7–26). Two patients started carbamazepine and prednisone on the same day (Patient 1 and 6). Maintenance daily doses of carbamazepine range between 100 mg and 600 mg. We started with 100 mg per day and increased 100 mg every 3–5 days until there was good seizure control or adverse drug reactions, such as somnolence or dizziness. When patients reached seizure freedom, we stopped increasing the dose.

We suggest a standard 1 mg/Kg/day of oral prednisone, for about 2–4 weeks, with a slow tapering taking into account the following immunotherapy (e.g. rituximab would need a shorter period (1 month), compared to azathioprine or mycophenolate mofetil (2–3 months).

Four patients received azathioprine as a steroid-sparing drug, 1 patient received mycophenolate (Patient 3, due to liver enzyme elevation with azathioprine) and 1 patient received rituximab (Patient 2).

Two patients reported allergy, Patient 3 developed a Steven Johnson Syndrome after 2.5 weeks after repeated iodinated contrast and 3 weeks after carbamazepine initiation; Patient 6 developed a mild rash 4 weeks after carbamazepine initiation (he achieved seizure freedom after 10 days), changing his AED to oxcarbazepine plus lacosamide with only suboptimal response.

## 4 Discussion

The present case-series highlights the clinical presentation of six patients with LGI1 -antibody mediated seizures diagnosed and treated mainly within outpatient based clinical care. The recognition of FBDS and piloerection seizures, pathognomonic of this entity, allowed a correct autoimmune and paraneoplastic workup, as well as early treatment with immunotherapy. In some patients, a response to low-dose carbamazepine was observed within 2 weeks of treatment initiation and sustained after immunosuppressive treatment. We also observed that early seizure cessation and treatment with steroid-sparing immunosuppressants was able to prevent the development of cognitive impairment, usually related to this disease.

Different percentages of seizure freedom have been described with varied combinations of antiepileptic drugs and immunotherapy, ranging from 51% to 89% (Thompson, 2018), (Feyissa et al., 2017) (de Bruijn et al., 2019). A recent randomized placebo-controlled trial of intravenous immunoglobulin (IVIG) showed that 6/8 patients in the IVIG group were responders (50% reduction in seizure frequency from baseline to 5 weeks) compared to 2/9 in the placebo group. Seizure freedom was achieved in 2/8 (25%) in the IVIG group compared to 1/9 (11%) in the placebo arm. Notably, only 3/8 patients in the IVIG group and 2/9 in the placebo group received sodium blocking channel antiepileptic drugs (Dubey et al., 2020).

Similar to our results, the effectiveness of carbamazepine in patients with LGI1 antibody mediated seizures has been suggested by Feyissa et al. and de Bruijn et al. We propose the possibility of synergism in combining low dose carbamazepine with early prednisone to account for the rapid complete response in our cohort (median 13 days to seizure freedom). This is despite the longer delay between seizure onset and the start of antiepileptic drugs compared to de Bruijn et al. (164 vs. 64 days), and the use of a monotherapy approach, also different to de Bruijn et al. (median of 2 antiepileptic drugs (range 0–9)) and Feyissa et al. (median of 2 (range 1–6)). In the study of de Bruijn et al., of patients with LGI1 antibody mediated seizures treated with both levetiracetam and carbamazepine (*n* = 15), carbamazepine appeared to be more effective (*p* = .031) (de Bruijn et al., 2019). While in the study of Feyizza et al., none of the patients became seizure-free using levetiracetam, and seizure freedom was only achieved after carbamazepine, lacosamide, phenytoin or oxcarbazepine (Feyissa et al., 2017). On the other hand, in a study by Thompson et al. only 10% of patients achieved seizure freedom after antiepileptic drugs alone (median 186 days, IQR 7–274). Whereas, 88% achieved seizure freedom after combination of antiepileptic drugs and immunotherapy after 90 days, and three patients achieved seizure freedom after immunotherapy alone (after 2 days in 2 patients, and after 14 days in 1 patient) (Thompson et al., 2018). The aforementioned variability could be explained by different disease severity/phenotypes, but also highlights the need for early identification of LGI1 antibody mediated seizures and prompt treatment, probably with a combination of sodium channel blockers and immunotherapy.

Considering seizure control as the overall goal, it is important to note that the gold standard for registering seizures, especially subclinical, is prolonged inpatient video-EEG monitoring. Nonetheless, the ictal registry in this group of patients is low (ictal EEG changes accompanied only 5/86 in FBDS and 18/53 subclinical seizures), interictal epileptiform discharges can be observed in only 25%, and slow-wave activity in 69% (Aurangzeb et al., 2017). In this context, we think that a detailed evaluation of the seizure semiology could be suitable for outpatient assessment and treatment monitoring.

Results from cognitive assessment suggest short-term memory complaints are one of the most common symptoms in patients with LGI1-antibody mediated disease. Additionally, our results suggest that successful treatment prevents further cognitive decline, although evidence of cognitive improvement should be confirmed with larger studies with a longer follow-up.

Since antibody titers are yet not established to accurately monitor the clinical course, some authors suggest a comprehensive approach including seizure control, neuropsychological evaluation, and even MRI metrics, such as brain atrophy or white matter integrity (Yelam et al., 2019; Szots et al., 2017). Nonetheless, the latter seems difficult to implement, mainly due to the current barriers of individual brain atrophy measurements (e.g. technical variability and biological confounding factors, such as pseudoatrophy and dehydration), similar to those observed in other immune-mediated disorders such as Multiple Sclerosis (Sastre-Garriga et al., 2020).

A higher incidence of drug induced allergic reactions have been reported (32%–35%), and some may relate to HLA-A*31:01 (Thompson et al., 2018). Two of our patients (33%) presented with allergy during treatment with carbamazepine, in line with previous reports. Also, it is important to consider the increased risk of drug interactions, especially in elderly patients with polypharmacy. This highlights the necessity for slow titration and early detection, as well as the need for finding the best treatment scheme for these patients.

Limitations of the present case-series are the small sample size with rather short follow-up time, and with longer time of diagnostic delay than previously reported in larger international cohorts. From a cognitive standpoint, a more extensive prospective study with formal neuropsychological testing and follow-up is also required to clarify the cognitive effects of LGI1 antibody mediated disease and its response to treatment.

In conclusion, early identification of LGI1-antibody mediated seizures and timely treatment with combination therapy, for example, with low dose carbamazepine and prednisone, could be a cost-effective protocol for outpatient clinical care in developing countries with limited resources.

The following are the supplementary data related to this article.

Supplementary Table 1 Cognitive performance. Supplementary Table 1

## Funding

SRI is supported by the 10.13039/100010269Wellcome Trust (104079/Z/14/Z), the 10.13039/501100000769UCB-Oxford University Alliance, BMA Research Grants—Vera Down grant (2013) and Margaret Temple (2017) and 10.13039/501100000295Epilepsy Research UK (P1201). The research was funded/supported by the 10.13039/501100000272National Institute for Health Research (NIHR) Oxford Biomedical Research Centre [(BRC); the views expressed are those of the author(s) and not necessarily those of the NHS, the NIHR or the Department of Health].

## Declaration of Competing Interest

RUSM, EC, RS, CC, AM, JPC, BS, MCM, and MH nothing to disclose. SRI is a coapplicant and receives royalties on patent application WO/2010/046716 (U.K. patent no., PCT/GB2009/051441) entitled’Neurological Autoimmune Disorders'. The patent has been licensed for the development of assays for LGI1 and other VGKC-complex antibodies. He has received honoraria from UCB, MedImmun, ADC therapeutics and Medlink Neurology.
