# Supplementary material for: LGI1-antibody associated epilepsy successfully treated in the outpatient setting
Source: J Neuroimmunol. 2020 Aug 15;345:577268. doi: 10.1016/j.jneuroim.2020.577268 (PMC7339132; doi:10.1016/j.jneuroim.2020.577268)
Supplement: Supplementary Table 1 — Cognitive performance. [file mmc1.docx]

**Supplementary Table. Cognitive performance**

| **Patient 1** | **Summary of the first cognitive symptoms reported at disease onset:** | | | | |
| --- | --- | --- | --- | --- | --- |
|  | *Short-term memory problems, inattention and disinhibition* | | | | |
|  |  |  | **First assessment** | **Follow-up 1** | **Follow-up 2** |
|  | **Time from symptom onset** | | 3 months | 9 months | 16 months |
|  | **Time from treatment onset** | | months |  |  |
|  | **Time seizure free** |  | months |  |  |
|  | **Patient`s complaint** | Cognitive | memory | memory | none |
|  |  | Psychiatric | none | none | none |
|  | **Collateral history** | Cognitive | memory | memory | none |
|  |  | Psychiatric | disinhibition | none | none |
|  | **Functionality** | ADL | independent | independent | independent |
|  |  | IADL | dependent | independent | independent |
|  | **MoCA** | Total score | 27/30 | 28/30 | 30/30 |
|  |  | Trail B | 1/1 | 1/1 | 1/1 |
|  |  | Cube copy | 1/1 | 1/1 | 1/1 |
|  |  | Clock drawing | 3/3 | 3/3 | 3/3 |
|  |  | Naming | 3/3 | 3/3 | 3/3 |
|  |  | Digit span forward | 1/1 | 1/1 | 1/1 |
|  |  | Digit span backward | 1/1 | 0/1 | 1/1 |
|  |  | Letter A cancellation | 1/1 | 1/1 | 1/1 |
|  |  | Serial sevens | 3/3 | 3/3 | 3/3 |
|  |  | Repetition | 2/2 | 2/2 | 2/2 |
|  |  | Verbal fluency (F words) | 0/1 (7) | 1/1 (16) | 1/1 (16) |
|  |  | Similarities | 2/2 | 2/2 | 2/2 |
|  |  | Free recall | 3/5 | 4/5 | 5/5 |
|  |  | Cued recall | 5/5 | 5/5 |  |
|  |  | Multiple options |  |  |  |
|  |  | Orientation | 6/6 | 6/6 | 6/6 |
|  | **FAB** | Total score | 14/18 | 15/18 | 18/18 |
|  |  | Similarities | 2/3 | 3/3 | 3/3 |
|  |  | Verbal fluency (A) | 2/3 (7) | 3/3 (11) | 3/3 (15) |
|  |  | Luria test | 1/3 | 3/3 | 3/3 |
|  |  | Sensitivity to interference | 3/3 | 3/3 | 3/3 |
|  |  | Inhibitory control | 3/3 | 0/3 | 3/3 |
|  |  | Prehension behaviour | 3/3 | 3/3 | 3/3 |
| **Patient 4** | **Summary of the first cognitive symptoms reported at disease onset:** | | | |  |
|  | *Short-term memory problems, spatial disorientation, irritability and insomnia* | | | |  |
|  |  |  | **First assessment** | **Follow-up 1** | **Follow-up 2** |
|  | **Time from symptom onset** | | 6 months | 10 months | - |
|  | **Time from treatment onset** | | 3 months | 7 months | - |
|  | **Time seizure free** |  | 3 months | 7 months | - |
|  | **Patient`s complaint** | Cognitive | memory | none | - |
|  |  | Psychiatric | none | none | - |
|  | **Collateral history** | Cognitive | memory | none | - |
|  |  | Psychiatric | irritability | mild irritability | - |
|  | **Functionality** | ADL | independent | independent | - |
|  |  | IADL | dependent | independent | - |
|  | **MoCA** | Total score | 21/30 | 24/30 | - |
|  |  | Trail B | 1/1 | 1/1 | - |
|  |  | Cube copy | 1/1 | 1/1 | - |
|  |  | Clock drawing | 3/3 | 3/3 | - |
|  |  | Naming | 3/3 | 3/3 | - |
|  |  | Digit span forward | 0/1 | 0/1 | - |
|  |  | Digit span backward | 1/1 | 1/1 | - |
|  |  | Letter A cancellation | 1/1 | 1/1 | - |
|  |  | Serial sevens | 2/3 | 2/3 | - |
|  |  | Repetition | 0/2 | 0/2 | - |
|  |  | Verbal fluency (F words) | 1/1 (14) | 1/1 (14) | - |
|  |  | Similarities | 2/2 | 2/2 | - |
|  |  | Free recall | 1/5 | 3/5 | - |
|  |  | Cued recall | 4/5 | 5/5 | - |
|  |  | Multiple options | 5/5 |  | - |
|  |  | Orientation | 5/6 | 6/6 | - |
|  | **FAB** | Total score | 13/18 | 15/18 | - |
|  |  | Similarities | 3/3 | 3/3 | - |
|  |  | Verbal fluency (A) | 3/3 (13) | 3/3 (16) | - |
|  |  | Luria test | 1/3 | 2/3 | - |
|  |  | Sensitivity to interference | 3/3 | 3/3 | - |
|  |  | Inhibitory control | 0/3 | 1/3 | - |
|  |  | Prehension behaviour | 3/3 | 3/3 | - |
| **Patient 5** | **Summary of the first symptoms reported at disease onset:** | | |  |  |
|  | *Short-term memory problems* | | |  |  |
|  |  |  | **First assessment** | **Follow-up 1** | **Follow-up 2** |
|  | **Time from symptom onset** | | 8 months | - | - |
|  | **Time from treatment onset** | | 1 month | - | - |
|  | **Time seizure free** |  | 4 days | - | - |
|  | **Patient’s complaint** | Cognitive | memory | - | - |
|  |  | Psychiatric | none | - | - |
|  | **Collateral history** | Cognitive | memory | - | - |
|  |  | Psychiatric | none | - | - |
|  | **Functionality** | ADL | independent | - | - |
|  |  | IADL | independent | - | - |
|  | **MoCA** | Total score | 23/30 | - | - |
|  |  | Trail B | 1/1 | - | - |
|  |  | Cube copy | 1/1 | - | - |
|  |  | Clock drawing | 3/3 | - | - |
|  |  | Naming | 2/3 | - | - |
|  |  | Digit span forward | 1/1 | - | - |
|  |  | Digit span backward | 1/1 | - | - |
|  |  | Letter A cancellation | 1/1 | - | - |
|  |  | Serial sevens | 2/3 | - | - |
|  |  | Repetition | 0/2 | - | - |
|  |  | Verbal fluency (F words) | 1/1 (20) | - | - |
|  |  | Similarities | 2/2 | - | - |
|  |  | Free recall | 2/5 | - | - |
|  |  | Cued recall | 3/5 | - | - |
|  |  | Multiple options | 5/5 | - | - |
|  |  | Orientation | 6/6 | - | - |
|  | **FAB** | Total score | 16/18 | - | - |
|  |  | Similarities | 3/3 | - | - |
|  |  | Verbal fluency (A) | 3/3 (11) | - | - |
|  |  | Luria test | 3/3 | - | - |
|  |  | Sensitivity to interference | 2/3 | - | - |
|  |  | Inhibitory control | 2/3 | - | - |
|  |  | Prehension behaviour | 3/3 | - | - |
| **Patient 6** | **Summary of the first symptoms reported at disease onset:** | | |  |  |
|  | *Short-term memory problems and mild confusion.* | | |  |  |
|  |  |  | **First assessment** | **Follow-up 1** | **Follow-up 2** |
|  | **Time from symptom onset** | | 8 months | - | - |
|  | **Time from treatment onset** | | 3 months | - | - |
|  | **Time seizure free** |  | 3 months | - | - |
|  | **Patient’s complaint** | Cognitive | memory | - | - |
|  |  | Psychiatric | none | - | - |
|  | **Collateral history** | Cognitive | memory | - | - |
|  |  | Psychiatric | apathy | - | - |
|  | **Functionality** | ADL | independent | - | - |
|  |  | IADL | independent | - | - |
|  | **MoCA** | Total score | 28/30 | - | - |
|  |  | Trail B | 1/1 | - | - |
|  |  | Cube copy | 1/1 | - | - |
|  |  | Clock drawing | 3/3 | - | - |
|  |  | Naming | 3/3 | - | - |
|  |  | Digit span forward | 1/1 | - | - |
|  |  | Digit span backward | 1/1 | - | - |
|  |  | Letter A cancellation | 1/1 | - | - |
|  |  | Serial sevens | 3/3 | - | - |
|  |  | Repetition | 2/2 | - | - |
|  |  | Verbal fluency (F words) | 1/1 (17) | - | - |
|  |  | Similarities | 2/2 | - | - |
|  |  | Free recall | 4/5 | - | - |
|  |  | Cued recall | 5/5 | - | - |
|  |  | Multiple options |  | - | - |
|  |  | Orientation | 5/6 | - | - |
|  | **FAB** | Total score | 18/18 | - | - |
|  |  | Similarities | 3/3 | - | - |
|  |  | Verbal fluency (A words) | 3/3 (17) | - | - |
|  |  | Luria test | 3/3 | - | - |
|  |  | Sensitivity to interference | 3/3 | - | - |
|  |  | Inhibitory control | 3/3 | - | - |
|  |  | Prehension behaviour | 3/3 | - | - |
| MoCa Montreal Cognitive Assessment; FAB Frontal Assessment Battery | | | | | |
